# Medical Obituary

**Published:** 1853-12

**Authors:** 


### MEDICAL OBITUARY.

Bransby Cooper, F. R. S., died at London, on the 19th of August. This able and distinguished Surgeon, was a son of Rev. Samuel Cooper, who was elder brother of the eminent Sir Astley. Bransby was born Sept. 2d, 1792—entered the British service, as midshipman, at an early age, but soon left it, and by advice of his uncle, Sir Astley, spent two years ot the Norwich Hospital, then came to London and devoted himself to surgery. In 1812 he entered the army, as Assistant Surgeon, and went to the Peninsula, the seat of war. He has since occupied a prominent position as a surgeon, and his reputation is known to the world. He was the author of Surgical Essays, Lectures, and edited the Biography of Sir Astley Cooper. His death was sudden and remarkable. He was crossing the Hall of the Atheneum Club, when he stopped, called for a glass of water, and, before it was brought, fell dead, the blood spouting from his mouth. An autopsy indicated that the hemorrhage probably proceeded from a perforation of the right lingual artery and the result of ulceration. He was a kind and amiable man, much beloved and respected.

